# Effectiveness of Debriefing in Hybrid Simulation For Communication Skills Training in End-Of-Life Care

**DOI:** 10.1186/2197-425X-3-S1-A39

**Published:** 2015-10-01

**Authors:** CW Yang

**Affiliations:** National Taiwan University Hospital, Taipei, Taiwan, Province of China

## Introduction

Communication skills with patients and family are getting more and more important in intensive care units (ICUs), especially for end-of-life conditions. Hybrid simulation (combined high-fidelity simulators and standardized patients) training was put more and more emphases for communication skills in end-of-life care.

## Objectives

This randomized controlled study conducted in National Taiwan University Hospital (NTUH) were to explore learning effectiveness of simulation exposure and debriefing.

## Methods

In NTUH, all first-year internal medicine residents were all invited to attend the hybrid simulation training in end-of-life care before they started their clinical rotation in ICUs and randomized into two groups. There were two scenarios simulating end-of-life cases in ICUs for each trainee. For each scenario, trainees were exposed to a 20-minute high-fidelity simulator-based simulation of end-of-life cases, followed by encountering a 10-minute communication with patients' family who are played by trained actors. For experimental group, they received audiovisual assisted debriefing about communication skills for end-of-life care. For controlled group, they received only discussion about the performance during manikin-based high-fidelity simulation. Standardized communication skills assessment form was applied to evaluate each trainee during second encounter by family who were played by trained actors.

## Results

During 2011-2014, there were totally 93 residents completing the courses and included for study. Among them, 47 were experimental group, and 46 were controlled group. “Global rating” of communication skills were higher in experimental group (4.09 vs. 3.70, p < 0.05). Trainees of experimental group also got higher scores in aspect of “appropriate listening skills with minimal interruption to family” (4.09 vs. 3.78. p < 0.05).

## Conclusions

Hybrid simulation followed by debriefing is more effective than hybrid simulation alone in communication skills training for end-of-life care.

